# Cavity preparation with Er:YAGvs, Nd:YAG and diode lasers on remaining dentin thickness (RDT)

**DOI:** 10.6026/973206300212829

**Published:** 2025-08-31

**Authors:** Prajakta Ganesh Chougule, Manoj Kumar Meena, Abhishek Mishra, Mushir Mulla, Chetna Joshi, Tapapriya Nandi, Ankur Kakkad, Munaz Mulla

**Affiliations:** 1 Department of conservative Dentistry and Endodontics, BharatiVidyapeet, (Deemed to be University) Dental College & Hospital, Sangli, Maharastra, India; 2 Department of Prosthodontics, Daswani Dental College and Research Centre, Kota, Rajasthan, India; 3 Department of Oral & Maxillofacial Surgery, ITS Dental College, Greater Noida, India; 4 Department of Public Health, College of Applied Medical Sciences, Qassim University, Saudi Arabia; 5 Department of Periodontics, Seema Dental College and Hospital, Rishikesh, Uttarakand, India; 6 Intern, Kalinga Institute of Dental Science, Kalinga Institute of Industrial Technology (KIIT) Deemed to be University, Patia, Bhubaneswar, Odisha, India; 7 Department of Oral Medicine and Radiology, Hitkarini Dental College, Jabalpur, Madhya Pradesh, India; 8 Department of Periodontics, Saveetha Institute of Dental College and Hospitals, Saveetha Institute of Medical and Technical Sciences, Saveetha University, Chennai, Tamilnadu, India

**Keywords:** Er:YAG laser, Nd:YAG laser, diode laser, remaining dentin thickness, thermal damage, *in vitro* study

## Abstract

Preservation of remaining dentin thickness (RDT) during cavity preparation is crucial for maintaining pulpal health. Laser technology has revolutionized modern dentistry by offering precise, minimally invasive treatment. Therefore, it is of interest to compare the effects of Er:YAG, Nd:YAG and diode lasers on remaining dentin thickness (RDT) and thermal damage during cavity preparation under standardized laboratory conditions.A total of 150 extracted human premolars were randomly divided into three groups. Pre- and post-operative RDT was assessed using a stereomicroscope and temperature rise was recorded with pulp chamber thermocouples. Histological evaluation with hematoxylin and eosin staining was performed to assess thermal effects. Er:YAG laser demonstrated superior conservation of dentin and minimal thermal damage, making it optimal for hard tissue applications. Nd:YAG and diode lasers exhibited higher thermal effects and reduced dentin preservation, suggesting caution in their use near the pulp.

## Background:

Laser technology has revolutionized modern dentistry by offering precise, minimally invasive treatment options and improved patient outcomes [1]. Its application in hard tissue procedures, particularly in cavity preparation, has been extensively studied due to its potential advantages in reducing pain and preserving tooth structure [2, 3]. However, the interaction of different laser systems with dentin varies considerably, largely owing to differences in their wavelength and energy absorption characteristics [4, 5]. The preservation of remaining dentin thickness (RDT) during cavity preparation is crucial for maintaining pulpal health. Excessive removal of dentin or thermal damage from laser irradiation can compromise pulp vitality and long-term prognosis [6, 7]. Among the commonly used lasers, Er:YAG exhibits high absorption in water and hydroxyapatite, enabling effective ablation with minimal thermal diffusion [8]. Conversely, Nd:YAG and diode lasers, which are absorbed more by pigments and less by water, may generate greater thermal rise, increasing the risk of collateral damage [9, 10]. Previous studies have explored individual laser systems for dentinal tubule sealing and their thermal effects on dental tissues [1, 6 and 7]. Aghayan *et al.* (2021) compared diode, Nd:YAG and Er:YAG lasers with fluoride for dentinal tubule obstruction [1]. Gholami *et al.* (2011) highlighted variable occluding effects of different lasers on dentinal tubules *in vitro* [6]. Similarly, Umana *et al.* (2013) reported preliminary findings on diode lasers achieving tubule sealing but raised concerns over thermal implications [7]. However, a direct comparative analysis of Er:YAG, Nd:YAG and diode lasers for their effect on RDT and thermal damage under standardized settings remains limited. Therefore, it is of interest to compare the gap by evaluating and comparing the effects of Er:YAG, Nd:YAG and diode lasers on RDT and thermal changes in extracted human teeth.

## Materials and Methods:

This *in vitro* study utilized 150 non-carious, freshly extracted human premolars free of cracks, restorations, or structural defects. Teeth were disinfected and stored in 0.1% thymol solution at 4°C until experimentation, following ethical approval from the institutional review board. The samples were randomly allocated into three groups (n=50 per group) based on the laser system employed: Group A (Er:YAG laser, 2940 nm), Group B (Nd:YAG laser, 1064 nm) and Group C (diode laser, 980 nm). Standardized Class I cavities measuring 3 mm in depth and 3 mm in diameter were prepared on the occlusal surfaces using the respective lasers under controlled parameters. The Er:YAG laser was operated at 300 mJ energy, 10 Hz frequency and 250 µs pulse duration with air-water spray cooling, while the Nd:YAG and diode lasers were set at 2 W and 1.5 W in continuous wave mode (CW) respectively, both using air cooling. Remaining dentin thickness (RDT) was assessed pre- and post-operatively using a stereomicroscope at 20 x magnifications, with micro-CT imaging performed on selected samples for validation. Thermal changes during cavity preparation were monitored using Type-K thermocouples inserted into the pulp chambers and a temperature rise exceeding 5°C was considered potentially harmful to pulp tissue. Additionally, histological evaluation of sectioned samples stained with hematoxylin and eosin (H & E) was conducted to detect thermal effects such as charring and carbonization. Data were statistically analyzed using SPSS version 25, employing one-way ANOVA followed by post-hoc Tukey tests, with significance set at p<0.05.

## Results:

The comparison of remaining dentin thickness (RDT) among the three laser groups revealed significant differences. Group A (Er:YAG) demonstrated the highest mean RDT of 2.15 ± 0.12 mm, followed by Group B (Nd:YAG) with 1.85 ± 0.15 mm and Group C (Diode) with the lowest mean RDT of 1.68 ± 0.13 mm (Table 1 - see PDF). Statistical analysis using one-way ANOVA confirmed a highly significant difference between groups (p < 0.001). Post-hoc Tukey tests indicated that all pairwise comparisons between the groups were statistically significant (p < 0.05), with Er:YAG preserving significantly more dentin than both Nd:YAG and diode lasers.

The comparison of remaining dentin thickness (RDT) and thermal rise among the three laser groups demonstrated statistically significant differences. Remaining Dentin Thickness: Group A (Er:YAG) showed the greatest preservation of dentin with a mean RDT of 2.15 ± 0.12 mm, followed by Group B (Nd:YAG) at 1.85 ± 0.15 mm and Group C (Diode) at 1.68 ± 0.13 mm (Table 1 - see PDF). One-way ANOVA revealed significant differences between the groups (p < 0.001). Post-hoc Tukey tests confirmed that Er:YAG preserved significantly more dentin than both Nd:YAG and diode lasers (p < 0.05).Temperature Rise: Thermal analysis revealed that Group A (Er:YAG) exhibited the lowest mean temperature rise at 2.8 ± 0.4 ° C. Group B (Nd:YAG) showed the highest thermal rise (7.2 ± 0.6 ° C), followed by Group C (Diode) at 5.9 ± 0.5 ° C (Table 2 - see PDF). ANOVA showed these differences to be highly significant (p < 0.001). Pairwise comparisons indicated that Er:YAG caused significantly less thermal rise compared to Nd:YAG and diode lasers (p < 0.05).Histological evaluation corroborated these findings, with minimal thermal damage (absence of charring or carbonization) observed in Er:YAG-treated samples, while Nd:YAG and diode groups displayed varying degrees of carbonization and thermal alteration in dentinal tissue. Er:YAG group showed clean ablation with minimal thermal effects, Nd:YAG samples demonstrated significant carbonization and charring, while diode-treated specimens revealed moderate thermal damage.

## Discussion:

The present study compared the effects of Er:YAG, Nd:YAG and diode lasers on remaining dentin thickness (RDT) and thermal rise during cavity preparation, highlighting significant differences among the three systems. Er:YAG laser preserved the highest RDT and exhibited minimal thermal rise, while Nd:YAG and diode lasers showed greater dentin removal and higher temperature elevations. These findings align with Aghayan *et al.* who demonstrated that Er:YAG lasers, due to their high water absorption, provide superior hard tissue ablation with reduced thermal effects compared to Nd:YAG and diode lasers [1]. Preservation of dentin is critical for pulpal health, as thinner dentin can lead to hypersensitivity and compromised pulp vitality [2, 3]. In the current study, the mean RDT for Er:YAG (2.15 ± 0.12 mm) was significantly higher than Nd:YAG and diode groups. This supports Sgolastra *et al.* who reported the efficacy of Er:YAG in maintaining dentinal integrity during laser treatments [4]. Similarly, Doshi *et al.* observed minimal structural changes in dentin following low-level laser applications, which parallels our findings for Er:YAG [5]. Regarding thermal effects, Er:YAG's mean temperature rise (2.8 ± 0.4 °C) was well below the critical threshold of 5°C for pulpal damage. In contrast, Nd:YAG (7.2 ± 0.6 °C) and diode (5.9 ± 0.5 °C) exceeded this limit, raising concerns about pulpal safety. Gholami *et al.* confirmed the higher thermal interaction of Nd:YAG and diode lasers due to their deeper penetration and lower water absorption [6]. Umana *et al.* also highlighted diode lasers' moderate thermal effects on dentin, which is consistent with our results [7]. Chiga *et al.* emphasized that combining lasers with fluoride varnish can reduce permeability in eroded dentin, suggesting potential adjuncts for minimizing thermal risks [8]. Zapletalová *et al.* cautioned about pulpal injury with Nd:YAG lasers, reinforcing our observation of carbonization and charring in histological sections [9]. Aranha *et al.* similarly documented increased dentin permeability post-Nd:YAG irradiation [10]. Recent studies have extended these findings. Ahmed *et al.* reported that diode lasers combined with fluoride were effective in gamma-irradiated dentin, but thermal safety remained a concern [11]. Baghani *et al.* noted that Nd:YAG lasers showed mixed outcomes in dentin hypersensitivity treatment, partly due to thermal side effects [12]. Hoshyari *et al.* demonstrated that Er,Cr:YSGG lasers were superior in maintaining dentin integrity compared to diode lasers [13]. Yadahalli *et al.* found higher bond strength with chitosan-treated compared to chlorhexidine treated dentine specimens [14]. Öncü *et al.* further confirmed the varying effects of different lasers on dentinal tubules, highlighting Er:YAG's advantage in structural preservation [15]. Taken together, these studies support the present findings that Er:YAG laser offers significant clinical benefits in terms of dentin preservation and pulpal safety. Nd:YAG and diode lasers, while effective in certain applications, should be used cautiously near the pulp due to their higher thermal output. Clinically, the superior performance of Er:YAG suggests its preference for cavity preparations close to the pulp to avoid pulpal injury. While Nd:YAG and diode lasers remain excellent for soft tissue procedures, their use on hard tissue warrants caution due to thermal effects.

## Clinical relevance:

Selecting an appropriate laser system is crucial to optimize patient outcomes and minimize iatrogenic damage.

## Limitation:

This *in vitro* study may not fully replicate the clinical environment, as factors such as pulpal blood flow and patient variability were not considered.

## Future perspective:

Future *in vivo* studies are needed to validate these findings and optimize laser parameters for safe and effective clinical applications in cavity preparation.

## Conclusion:

Er:YAG lasers offer superior preservation of dentin and minimal thermal damage during cavity preparation, supporting their use in restorative dentistry. Nd:YAG and diode lasers, while effective, exhibited higher thermal effects and reduced dentin conservation, limiting their applicability for deep cavity preparations.
